# The role of HCN channels in peristaltic dysfunction in human ureteral tuberculosis

**DOI:** 10.1007/s11255-018-1816-y

**Published:** 2018-02-19

**Authors:** Fan He, Zhenxing Yang, Xingyou Dong, Zhenqiang Fang, Qian Liu, Xiaoyan Hu, Shanhong Yi, Longkun Li

**Affiliations:** Department of Urology, Xinqiao Hospital, the Third Military Medical University, No. 183 Xinqiao Main Street, Shapinba Dist., Chongqing, 400037 People’s Republic of China

**Keywords:** c-kit, HCN, Spontaneous contraction, Tuberculous ureter, ZD7288

## Abstract

**Objective:**

To explore the role of HCN channels in ureteral peristaltic dysfunction by comparing the changes in HCN channel levels between normal and tuberculous ureters.

**Methods:**

A total of 32 specimens of human upper ureters were collected by nephrectomy from patients with renal tumor (control group, *n* = 16) or from patients with renal tuberculosis (experimental group, *n* = 16); the two groups did not receive radiotherapy, chemotherapy, immunotherapy, or any other special treatment before the surgical procedure. An experimental study on smooth muscle strips of human upper ureters showed variation in contraction amplitude and frequency after adding ZD7288, a specific blocker of HCN channels. The expression of HCN channels in the ureter was confirmed by Western blot (WB) and by confocal analysis of double immunostaining for c-kit and HCN channel proteins.

**Results:**

Before the addition of ZD7288, the experimental and control groups showed significant differences in the frequency and amplitude of the spontaneous contraction of isolated ureteral smooth muscle strips. After ZD7288 was added, the frequency and amplitude of the contractions of the ureteral smooth muscle strips were significantly lower in both groups. The differences observed before and after ZD7288 treatment in each group were significant (*P* < 0.001), and the difference in contraction amplitude observed between the two groups before ZD7288 was also significantly different (*P* < 0.001). By using WB technology, we showed that the expression of HCN channels was present in normal human ureters, with the expression of HCN4 and HCN1 being the highest; the expression of HCN4 and HCN1 in the control and experimental groups were both statistically significant (*P* < 0.001). HCN4 and HCN1 were expressed in the mucosal and smooth muscle layers of human control ureters and tuberculous ureters, as revealed by a confocal analysis of double immunostaining for c-kit and HCNs proteins; there were significant differences between the two groups (*P* < 0.001).

**Conclusion:**

Four HCN channels are expressed in the ureter, mainly HCN4 and HCN1, suggesting that HCN channels are involved in the peristaltic contraction of ureteral ICCs, which may be an important reason for peristaltic dysfunction in ureteric tuberculosis.

## Introduction

The main physiological function of spontaneous contraction of the ureter is to promote continuous transport of urine to the bladder. Recently, studies have found that spontaneous peristaltic contraction disorder of the ureter is an important etiology of congenital giant ureter, congenital ureteropelvic junction (UPJ) deformity and non-obstructive hydronephrosis [1, 2]. The same clinical manifestations are also found in ureteral tuberculosis. According to previous research data, studies showed that the bladder in vitro had spontaneous contractile function and could inhibit the contractions after adding ZD7288, which is a special hyperpolarization-activated cyclic nucleotide-gated (HCN) channels blocker. Furthermore, urinary bladder interstitial cells of Cajal (ICCs) act as pacemaker cells to generate the physiological contraction of the bladder; the expression of HCN channels in ICCs is an important mechanism for generating spontaneous excitation–contraction in the bladder [3–12]. The peristaltic contraction of the ureter is similar to that of the bladder. Previous studies have shown that ICCs have a similar distribution and expression pattern in the ureter [13–16] compared to the bladder. Additionally, there was a correlation between the function of the ureter and the expression of ICCs in different pathological conditions [2, 15–19]. Therefore, if HCN channels were expressed in ureteral ICCs, could the variability in HCN channel expression impact the spontaneous contraction of the ureter under pathological conditions? To answer this question, we performed the following preliminary study.

## Materials and methods

### Clinical data

In vitro ureteral specimens from 32 cases of nephrectomy performed in our department of urology from Jul 2015 to Feb 2017 were divided into an experimental group (*n* = 16) and a control group (*n* = 16). All patients signed the relevant informed consent forms before the surgical procedure. The inclusion criteria for the control group were as follows: (1) preoperative diagnosis of renal tumor by CT or MRI, (2) unilateral renal tumor and normal contralateral kidney, (3) planned nephrectomy and (4) postoperative pathological diagnosis of clear cell renal cell carcinoma. The exclusion criteria for the control group were as follows: (1) preoperative treatment with radiotherapy, chemotherapy, immunotherapy or other special treatment and (2) postoperative diagnosis revealing the absence of renal clear cell carcinoma. The inclusion criteria for the experimental group were as follows: (1) clinical diagnosis of renal tuberculosis by chest radiography, tuberculin skin test, erythrocyte sedimentation rate, acid-fast staining of urine, IVP or CT, (2) unilateral kidney with no serious functional damage and contralateral normal kidney and (3) pathological diagnosis of caseous granuloma with visible Langhans giant cells and surrounded by lymphocytes and fibroblasts. The exclusion criteria for the experimental group were as follows: (1) preoperative treatment with radiotherapy, chemotherapy, immunotherapy or other special treatments, (2) postoperative pathological diagnosis showing no evidence of renal tuberculosis. The experimental group with preoperative diagnosis of renal tuberculosis comprised 8 males and 8 females; this composition was the same in the control group with preoperative diagnosis of renal tumors. The average age for the experimental group and the control group were 54.2 (± 4.2) and 56.8 (± 3.2) years, respectively. We confirmed that the patients were not receiving radiotherapy, chemotherapy or immunotherapy or other special treatments before surgery. All experiments involving clinical patient samples were performed in accordance with a protocol approved by the ethics committee of the Third Military Medical University.

### Ureter smooth muscle strip tension in vitro assay

As previously described, the ureter samples were obtained from nephrectomy. The ureters were carefully resected and freshly placed in ice-cold Kreb’s solution (containing the following components: 118.7 mM of NaCl, 1.2 mM of KH2PO4, 4.7 mM of KCl, 2.5 mM of CaCl2, 12.5 mM of NaHCO3, 1.2 mM of MgSO4, and 5. 5 mM of glucose). The ureter samples were longitudinally cut into strips of approximately 20 × 5 × 3 mm in dimension. Each strip was suspended vertically between two curved hooks and placed into a 10-ml organ bath that was filled with Kreb’s solution and maintained at 37 °C, 95% O_2_, pH 7.4. The upper hook was connected to a movable stretch transducer, and the lower hook was fixed to the bottom of the bath. After an equilibration period of 30 min, the strips were stretched gradually until the stretch load was maintained at 3 g; a cumulative concentration of 50 μM of the HCN blocker, ZD7288, was used to assess the effects of HCN channel inhibition on the spontaneous phasic contraction of the strips. The dynamic curves were recorded continuously with isometric force transducers and visualized with the signal acquisition system RM6280C (Chengyi Co., Chengdu, China).

### Western blot analysis

The total protein was isolated from ureters using the RIPA lysis buffer (Beyotime, Haimen, China), and the protein concentrations were measured using the Bio-Rad protein assay (Bio-Rad Laboratories, Hercules, CA). A total of 80 μg of protein extract was electrophoresed on SDS-PAGE and transferred onto polyvinylidene fluoride membranes (Merck Millipore, Billerica, USA). The membranes were incubated for 2 h in blocking buffer (5% bovine serum albumin dissolved in Tris-buffered saline solution) to prevent non-specific binding of the antibodies. Then, the membranes were incubated overnight at 4 °C with four HCN channel primary antibodies (purchased from Abcam, Cambridge, UK): mouse anti-HCN1 antibody (Abcam, ab84816, 1:1000), rabbit anti-HCN2 antibody (Abcam, ab126839, 1:500), rabbit anti-HCN3 antibody (Abcam, ab192025, 1:1000), mouse anti-HCN4 antibody (Abcam, ab69054, 1:1000), and with the rabbit anti-glyceraldehyde phosphate dehydrogenase (GAPDH) primary antibody (1:1500, Zhongshan, Peking, China). After being washed in Tris-buffered saline containing Tween (3 × 10 min), the membranes were incubated with horseradish peroxidase-conjugated anti-mouse or anti-rabbit IgG (1:5000, Zhongshan, Peking, China) for 2 h at room temperature. After incubating the membranes with the enhanced chemiluminescent substrate (ECL, Millipore, Billerica, MA), the protein bands were imaged and analyzed by the Molecular Imager ChemiDoc XRS System (Bio-Rad Laboratories). The band density was measured with the ImageLab software. The band density was normalized to the corresponding GAPDH loading control.

### Immunofluorescence staining

For the frozen section, the resected ureters were fixed in 4% paraformaldehyde solution for 2 h at 4 °C. After being washed in phosphate-buffered saline (PBS, 0.1 M, pH 7.4), the tissues were cryoprotected by immersion in 30% sucrose overnight at 4 °C. Then, the tissues were cut into 5-μm sections using a freezing microtome. Tissue sections were washed in PBS for 10 min and immersed in immunostaining blocking buffer (Beyotime, Haimen, China) for 30 min at room temperature to block non-specific binding sites on the tissue. The sections were incubated overnight at 4 °C with two HCN channel primary antibodies (purchased from Abcam, Cambridge, UK) and a c-kit antibody (Santa Cruz, Shanghai, China) including the following: mouse anti-HCN1 antibody (ab84816, 1:200), rabbit anti-HCN4 antibody (ab69054, 1:200) and goat anti-c-kit antibody (sc-1494 1:50). After rinsing in phosphate-buffered saline (PBS) (3 × 10 min), the sections were incubated with the following fluorescence-conjugated secondary antibodies for 1 h at room temperature: Alexa 488 goat anti-mouse IgG (1:200, A0428, Beyotime, Shanghai, China), Alexa Cy3 donkey anti-goat IgG (1:200, A0502, Beyotime, Haimen, China), Alexa 555 donkey anti-rabbit IgG (1:200, A0453, Beyotime, Shanghai, China) and Alexa 488 mouse anti-goat IgG (1:50, bs-0294 M-AF488, Bioss, Beijing, China). Next, the preparations were washed in PBS (3 × 10 min) and incubated with 4′,6-diamidino-2-phenylindole (DAPI, Beyotime, Haimen, China) to label the cell nucleus. In each run, negative controls (no primary antibody) were also included. The pictures were captured by laser confocal microscopy. The mean intensity value of fluorescence was calculated using the ZEN imaging software (Carl Zeiss AG, Jena, Germany).

### Statistical analyses

The experimental data are presented as the mean ± SD. Independent samples *t* tests or rank sum test were performed using the SPSS 16.0 software (SPSS Inc., Chicago, IL) for each comparison. All *t* tests were two-tailed, and all differences with *P* values below 0.05 (*P* < 0.05) were considered to be statistically significant.

## Results

### Effect of ZD7288 treatment on the contractility of ureteral smooth muscle strips isolated from the two groups (Fig. 1)

**Fig. 1:**
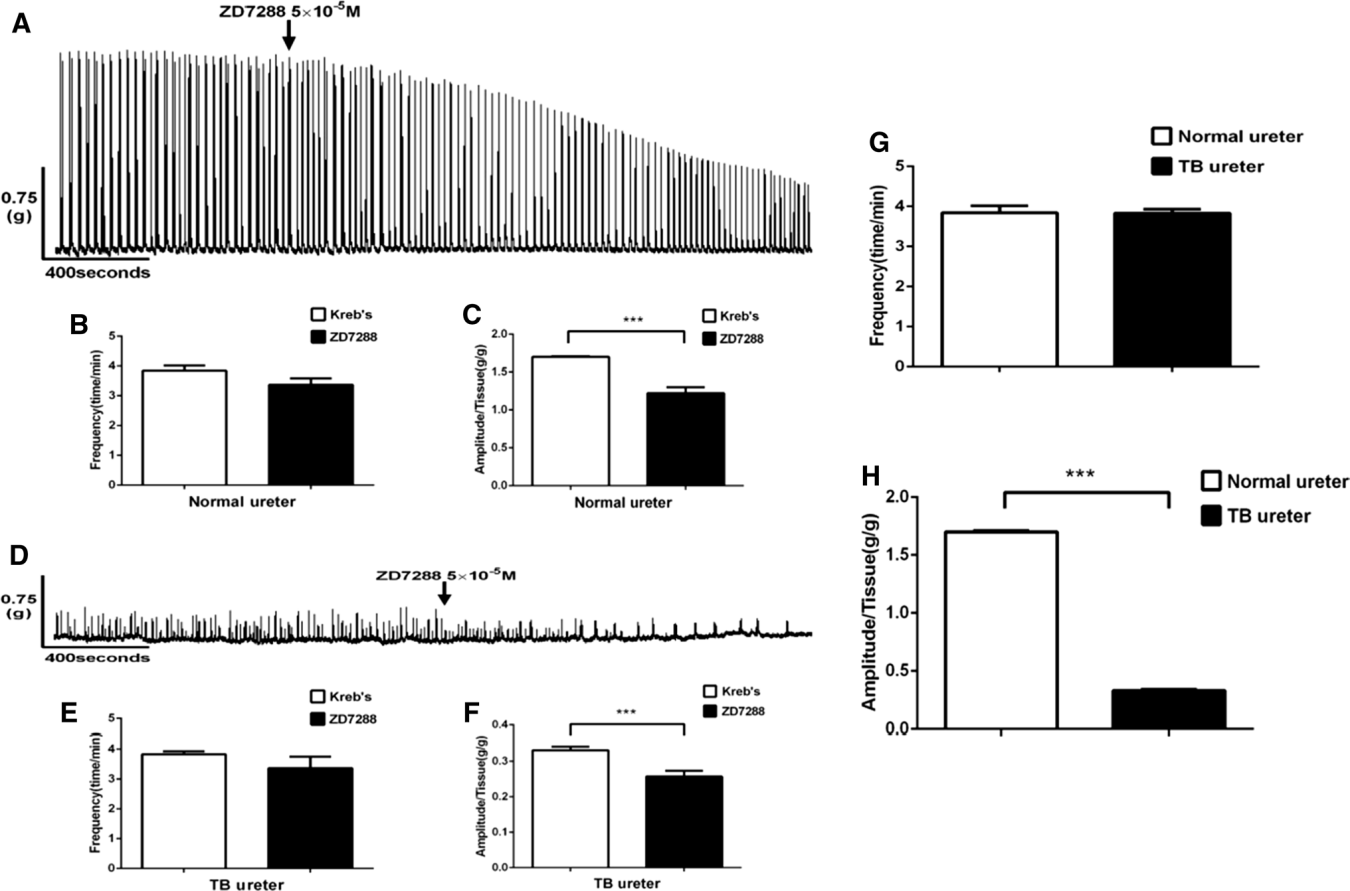
Effects of ZD7288 treatment on the spontaneous contraction of the ureteral smooth muscle strips. Tension recording shows the effects of ZD7288 treatment on the contractile activities of the ureteral smooth muscle strips in the control group (**a**, *n* = 6) and in the experimental group (**d**, *n* = 6). ***p < 0.001

To study the effects of ZD7288 treatment on the ureteral smooth muscle contractility, the ureter smooth muscle strip tension assay was performed with samples from the control group (Fig. 1a) and from the experimental group (Fig. 1d). For the statistics of the frequency and amplitude of contraction, we calculated the number of times of complete contraction waveform of 10 units in every 60 s to obtain ten groups of contraction frequency and contraction peak and the average value of contraction peak in 60 s as a unit within 10 min of steady contraction before successively dosing. The same statistical methods were applied before and after dosing. The ZD7288 treatment did not significantly change the contraction frequency in the control group (3.85 ± 0.17 vs. 3.37 ± 0.22, respectively, independent-sample *t* test, *p* = 0.116) (Fig. 1b) and in the experimental group (3.83 ± 0.11 vs. 3.37 ± 0.38, independent-sample *t* test, *p* = 0.215) (Fig. 1e), but it significantly changed the amplitude of spontaneous contraction in the control group (1.70 ± 0.01 vs. 1.22 ± 0.08, respectively, independent-sample *t* test, *p* < 0.001) (Fig. 1c) and in the experimental group (0.33 ± 0.01 vs. 0.26 ± 0.02, respectively, *p* < 0.001) (Fig. 1f). Comparing the control group with the experimental group, the frequency of spontaneous contraction was not significantly different (3.85 ± 0.17 vs. 3.83 ± 0.11, respectively, independent-sample *t* test, *P* = 0.946) (Fig. 1g); however, the amplitude of spontaneous contraction was significantly different (1.70 ± 0.01 vs. 0.33 ± 0.01, respectively, independent-sample *t* test, *P* < 0.001) (Fig. 1h).

### Analysis of the differences in HCN channel transcripts and protein levels between normal ureter and tuberculous ureter (Fig. 2)

**Fig. 2:**
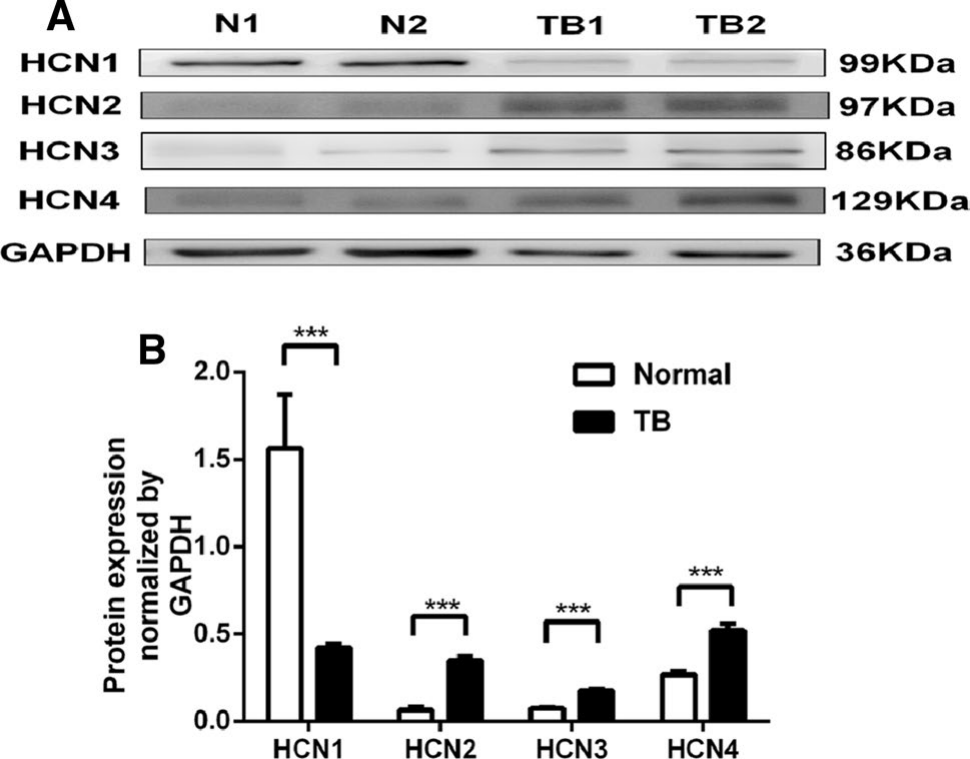
Differences in the protein expression level of four HCN subtypes between the two groups. A Western blot analysis of protein lysates from tuberculous ureters (TB, TB1, TB2) and control ureters (Normal, N1, N2) that are probed with anti-HCN1, anti-HCN2, anti-HCN3, anti-HCN4 antibodies and anti-GAPDH antibody, which serves as an endogenous loading control. The molecular weights of the four HCN subtypes and of GAPDH are 99, 97, 86, 129 and 36 kDa, respectively (**a**, *n* = 4). Tuberculosis increased the protein expression levels of three HCN channels (**b**), including HCN2, HCN3 and HCN4, and decreased the protein expression levels of HCN1. ***Independent-sample *t* test, *p* < 0.001

Using Western blot analysis to compare the human ureters from normal and tuberculosis cases, we assessed the expression of four HCN channel subtypes (Fig. 2a). In both groups, the two highest expression levels were obtained for HCN4 and HCN1. Tuberculosis decreased the expression of HCN1 (1.56 ± 0.31 vs. 0.42 ± 0.02, respectively, independent-sample *t* test, *P* < 0.001) and increased the expression of HCN2 (0.07 ± 0.02 vs. 0.35 ± 0.02, respectively, independent-sample *t* test, *P* < 0.001), HCN3 (0.07 ± 0.05 vs. 0.17 ± 0.01, respectively, independent-sample *t* test, *P* < 0.001) and HCN4 (0.27 ± 0.02 vs. 0.52 ± 0.04, respectively, independent-sample *t* test, *P* < 0.001) (Fig. 2b).

### Analysis of the distribution of HCN channels in human ureters from the two groups (Fig. 3)

**Fig. 3:**
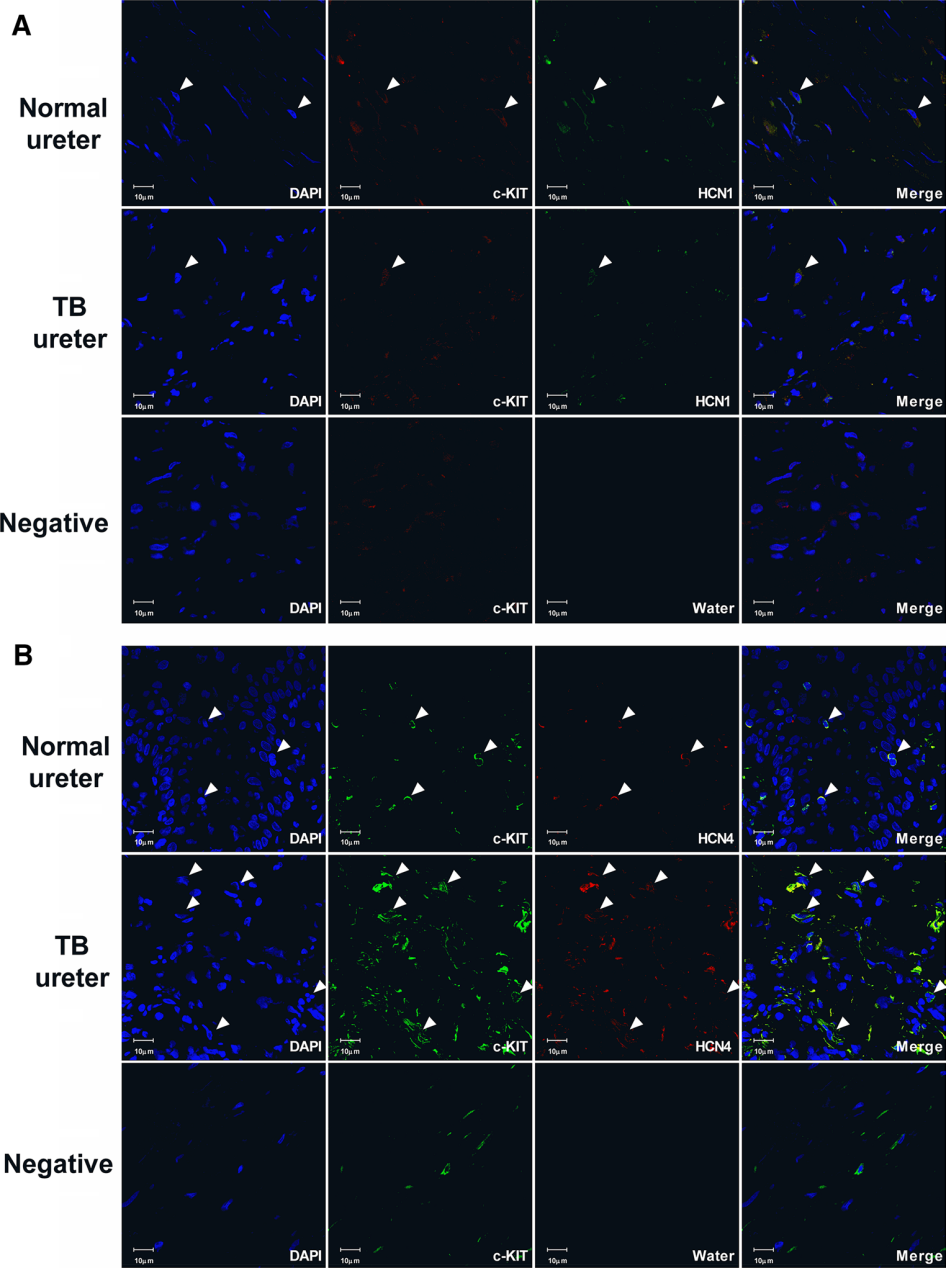
Immunofluorescence data reveal that HCN channels and c-kit protein co-label the suburothelium and the ureteral smooth muscle bundles. The white arrow indicates immunoreactive cells in the human ureter. Double immunofluorescence of HCN1 and c-kit is shown in **a**: 1, nuclei counterstained with DAPI(blue); 2, c-kit staining (red); 3, HCN1 staining (green); 4, merged image showing the co-localization of c-kit and HCN1. The negative control was incubated with water instead of anti-HCN1 to exclude non-specific staining (**a**). Double immunofluorescence of HCN4 and c-kit is shown in **b**: 1, nuclei counterstained with DAPI (blue); 2, c-kit staining (green); 3, HCN4 staining (red); 4, merged image showing the co-localization of c-kit and HCN4. The negative control was incubated with water instead of anti-HCN4 to exclude non-specific staining (**b**)

The analysis of the expression and distribution of HCN channels by immunofluorescence staining revealed that in both groups, the two major HCN channel subtypes (HCN1, HCN4) were mainly distributed in the lamina propria and detrusor muscularis with protein levels that were significant for both HCN1 (Fig. 3a) and HCN4 (Fig. 3b).

## Discussion

Ureteral tuberculosis, which develops secondarily to renal tuberculosis due to tuberculosis infection, produces specific inflammation that affects spontaneous peristaltic movement of the ureter, and in turn, these peristaltic changes increase the inflammation that eventually leads to renal atresia caused by ureteral fibrosis and results in irreversible loss of renal function. Ureteral peristaltic dysfunction, caused by the inflammation of tuberculosis, can cause non-obstructive hydronephrosis. Aggravated renal tuberculosis infection is an important factor leading to renal dysfunction. Therefore, ureteral peristaltic dysfunction is an important factor in ureteral tuberculosis that increases hydronephrosis and damages renal function. Ureteral peristaltic dysfunction is often a direct or indirect effect of increased renal lesion, which is common in inflammation, mechanical obstruction, congenital disorders of the urinary system and other diseases [1, 2, 4, 19]. Compared to normal ureteral peristalsis, ureteral peristaltic dysfunction is different in the sense that peristalsis frequency, creeping flow direction and creeping flow amplitude are abnormal, even if the obstruction is removed. Pathological changes of the ureter due to long-term obstruction can lead to reduced ureteral peristalsis or loss of ureteral peristalsis resulting in ureteral dynamic obstruction that continuously increases renal damage. Changes in the spontaneous pacing function of the ureter are one of the important factors affecting ureteral peristalsis. Therefore, exploring the molecular changes occurring in the ureter with the pacing function of ICCs in pathological contexts is an important way to understand the mechanism of ureteral spontaneous peristalsis dysfunction.

Interstitial cells of Cajal (ICCs) are a class of pacing cells, normally labeled with c-kit, that were found in the gastrointestinal tract by the Spanish anatomist Santiago Ramony Cajal in 1893; therefore, ICCs are also known as Cajal cells. Studies have shown that ICCs have the capacity to regulate the gastrointestinal motility. The functional distribution of ICCs and their structural abnormalities are an important cause of some gastrointestinal motility disorders such as achalasia and some congenital diseases [20–22]. The bladder and ureter are not impacted by spontaneous or excitatory nervous activity, unlike the gastrointestinal tract which also has an independent peristaltic function, and studies from the literature and from our group found that the urinary tract contains c-kit positive cells, with morphological and structural characteristics similar to gastrointestinal ICCs, that we called urinary tract ICC-like cells [3–9]. By analyzing ICCs from the bladder, we found that the distribution and function of ICCs is closely related to excitatory abnormalities of the bladder [7, 8]. In the inflamed bladder animal model, the expression and distribution of ICCs were significantly different [9, 23]. Studies of bladder ICCs and of the bladder pacing function have shown that the HCN channels present in ICCs play a pivotal role in the origin of the excitatory pathway of the bladder [9, 24]. Therefore, it is important to determine whether the spontaneous peristaltic contraction of the ureter uses an HCN channel-based ICCs pacemaker excitatory contraction pathway, such as the bladder.

HCN channels are non-selective cation channels that are voltage and cyclic adenosine monophosphate-regulated and that can produce specific Ih current; HCN channels are considered to be important markers of pacing cells [25, 26] and are widely expressed in the brain [27, 28] and heart [29–34]. The literature and our study confirmed the presence of HCN channels in the ureter [35, 36], but there is no direct evidence that HCN channels are expressed in ureteral ICCs. We identified the presence of HCN channels at the protein level in the ureter by RT-PCR and WB assays, specifically HCN4 and HCN1; the use of the HCN-specific blocker ZD7288 significantly inhibits the spontaneous contraction of the ureter in vitro. Immunofluorescence staining reveals that the ICC-specific marker c-kit and HCN4 and HCN1 are co-expressed in the ureteral submucosal and muscular layers. These results suggest that HCN channels are expressed in ureteral ICCs, which probably act as pacing cells for spontaneous excitatory contraction of the ureter; the core function of ICCs may be dependent upon an HCN channel-based pacing pathway. However, there is no direct evidence to confirm the HCN channels in the ureteral ICCs cells by the whole cell patch clamp technique. In addition, we lack animal experiments to prove the expression of this difference. This study only highlights the changes in HCN channels observed in the disease state of the ureter, but the mechanism that generates these differences remains unclear. We need to conduct further studies to elucidate this mechanism.
